# Effective Enrichment and Quantitative Determination of Trace Hg^2+^ Ions Using CdS-Decorated Cellulose Nanofibrils

**DOI:** 10.3390/nano10112218

**Published:** 2020-11-07

**Authors:** Hilal Ahmad, Ibtisam I. Bin Sharfan, Rais Ahmad Khan, Ali Alsalme

**Affiliations:** 1 Division of Computational Physics, Institute for Computational Science, Ton Duc Thang University, Ho Chi Minh City 700000, Vietnam; hilalahmad@tdtu.edu.vn; 2 Faculty of Applied Sciences, Ton Duc Thang University, Ho Chi Minh City 700000, Vietnam; 3 Department of Chemistry, College of Science, King Saud University, Riyadh 11451, Saudi Arabia; 437202977@student.ksu.edu.sa (I.I.B.S.); krais@ksu.edu.sa (R.A.K.)

**Keywords:** preconcentration, solid-phase extraction, mercury toxicity, nanoadsorbent, inductively coupled plasma optical emission spectroscopy

## Abstract

Water pollution caused by metal contamination is of serious concern. Direct determination of trace metal ions in real water samples remains challenging. A sample preparation technique is a prerequisite before analysis. Herein, we report the facile water-based hydrothermal synthesis of cadmium sulfide nanoparticles on a cellulose nanofiber surface to prepare a new adsorbent material. Field emission scanning electron microscopy, high-resolution tunneling electron microscopy, elemental mapping and X-ray photoelectron microscopy were used to characterize the surface morphology, structural determination, elemental composition and nature of bonding. The nanoadsorbent (cadmium-sulfide-decorated cellulose nanofibrils (CNFs@CdS)) was employed for the solid-phase extraction and determination of trace Hg(II) from aqueous media. The experimental conditions were optimized systematically and the data show a good Hg(II) adsorption capacity of 126.0 mg g^−1^. The CNFs@CdS adsorbent shows the selective removal of Hg(II) accordingly to the hard and soft acid–base theory of metal–ligand interaction. A high preconcentration limit of 0.36 µg L^−1^ was obtained with a preconcentration factor of 580. The lowest level of trace Hg(II) concentration, which was quantitatively analyzed by the proposed method, was found to be 0.06 µg L^−1^. No significant interferences from the sample matrix were observed in the extraction of Hg(II). Analysis of the standard reference material (SRM 1641d) was carried out to validate the proposed methodology. Good agreement between the certified and observed values indicates the applicability of the developed methodology for the analysis of Hg(II) in tap water, river water and industrial wastewater samples.

## 1. Introduction

Environmental water pollution is one of the leading causes of death worldwide, with an average of two million causalities per year due to contaminated drinking water [1,2]. The problem may worsen, as the World Health Organization (WHO) estimated that climate change will limit pure water access for half of the world’s population [3]. In a recent report, the United Nations (UN) stated that we could face a 40% water shortage in the near future [4]. In recent decades, an exponential increase in the usage of heavy metals in various industrial processes has caused their exposure to humans [5,6]. Modern constructions, industrial development and the untreated discharge of contaminated water into natural water sources are among the core reasons for environmental water pollution. Large amounts of metal-contaminated wastewater are discharged into the environment due to several industrial processes, such as electronics, electroplating, wood preservation and leather tanning. Long-term metal exposure to humans can occur during occupational activities, such as in mining and ore industries, mainly through inhalation and dermal routes, along with the consumption of metal-contaminated water and food and exposure to soil, dust and air.

Among heavy metal contamination, the high toxicity and prevalence of mercury (Hg(II)) are of great concern [7,8]. With no role in human homeostasis, it causes birth defects, multiple organ damage and is classified as carcinogenic to living systems [9]. In fact, long-term use of Hg(II) at trace-level concentrations leads to serious human health problems, including detrimental effects on the human brain, blood circulation and immune and reproductive systems [10,11,12]. A maximum permissible limit of 2.0 ppb of Hg(II) contamination was set by the US Environmental Protection Agency [5]. Thus, assessing such toxic metals is imperative for environmental monitoring, the remediation of toxic contaminants from water and human protection [13]. 

Sophisticated analytical tools for metal ion determination, such as atomic fluorescence spectrometry, graphite furnace atomic emission/absorption spectroscopy and inductively coupled plasma spectrometry, are common, with excellent features such as high sensitivity, a wide dynamic range and simultaneous multielement detection capabilities [14,15,16]. Direct analysis of natural samples for Hg(II) determination is difficult due to low analyte concentrations and high salt matrices, causing interferences and sampling limitations [17,18,19,20,21]. The enrichment/extraction of trace analyte has become essential for sample preparation and the improvement of instrument sensitivity, including solid-phase extraction (SPE), solvent extraction and coprecipitation techniques. Among them, SPE is the most common and widely accepted technique due to its simple operation, low cost, high enrichment factor, small sample requirement and the lack of a need for a harmful organic solvent. SPE has been used in both batch and column modes [22,23,24,25]. The adsorbent used in the SPE technique has undergone rapid growth and, recently, the use of nanomaterials (nanoadsorbents), such as graphene, carbon nanotubes, metal oxides, nanosilica and metal hydroxides, has emerged as the fastest-growing area in the field of separation science, especially in water quality monitoring and decontamination [23,26,27,28]. The high surface area of nanomaterials and ease of chemical modifications make them applicable in metal ion adsorption [29]. Such adequacies raise our interest in the exploration of metal chalcogenides for metal ion adsorption. Two-dimensional metal chalcogenides are porous and incomparable to metal oxides and have moved to the forefront of nanomaterial research [30,31]. Metal chalcogenides possess identical “Lewis soft” binding sites that provide high selectivity towards heavy metal ions. Cadmium sulfide (CdS) is a two-dimensional chalcogen, studied for its astounding physicochemical characteristics such as a high specific surface area and porous structure abundant in surface-active sites [32,33,34]. The use of bulk nanomaterials in SPE had led to column plugging and the loss of the nanoadsorbent under high flow rates. Herein, we immobilize CdS nanoparticles onto a cellulose nanofibrils (CNFs) surface to provide a solid support for improved and stable use in column operation compared to bulk. The smaller particle size uncovers more sulfur atoms than the bulk sample leading to the efficient adsorption of Hg(II) ions. These characteristics reveal that CdS-decorated CNFs (CNFs@CdS) could provide potential applications in SPE.

## 2. Experimental Section

### 2.1. Materials and Methods

#### 2.1.1. Materials

Cadmium acetate and sodium sulfide (99% purity) were purchased from Sigma-Aldrich (Steinem, Germany). A stock of divalent mercury ions of 1000 mg L^−1^ was purchased from Agilent (Melbourne, Australia) and used after successive dilutions. CNFs were obtained from Biocrown Biotechnology (Guangzhou, China) and sequentially washed with 5% HNO_3_ and deionized water (18.2 MΩ/cm) to remove any impurities, if present, before chemical functionalization.

#### 2.1.2. Synthesis of CNFs@CdS Nanoadsorbent

A 50.0 mmol amount of cadmium acetate and 43.8 mmol of sodium sulfide was mixed vigorously to make a homogenous solution in 100 mL of deionized water. The reaction mixture was transferred to a 250 mL Teflon-lined autoclave (Newstar company, Guangzhou, China) along with cellulose nanofibers (5 g). The autoclave was heated at 180 °C for 18 h under an air oven. The CdS-decorated cellulose nanofibers, after cooling to room temperature, were filtered off and sonicated in deionized water to wash weak/unbound particles. Finally, the product was filtered off and vacuum (oven)-dried at 60 °C before characterization and adsorption studies.

#### 2.1.3. Material Characterization

The prepared material’s surface morphology was characterized by field emission scanning electron microscope (FESEM, JSM-7800F, JEOL, Tokyo, Japan) and high-resolution tunneling electron microscopy (HRTEM, Tecnai G2 F30 S-TWIN 300 kV, Thermo Fisher, Hillsboro, OR, USA). For FESEM, the solid sample was placed onto a sample holder and carbon coating was carried out. For tunneling electron microscopy (TEM) analysis, the sample was dispersed in ethanol using a probe sonicator and deposited on a copper grid before TEM analysis. The elemental composition of CNFs@CdS was observed using energy-dispersive X-ray analysis (EDS, Bruker, Steinem, Germany) and X-ray photoelectron spectroscopy (XPS, Thermo Fisher, Melbourne, Australia) with an Mg K alpha source, a spot size of 500 µm and a 10 nm detection depth. The Brunauer–Emmett–Teller (BET) surface area measurements were carried out using an Autosorb-iQ one-station N_2_ gas sorption analyzer (Quantachrome Instruments, Boynton Beach, FL, USA). Inductively coupled plasma optical emission spectrometer (ICP-OES, Avio 200; Perkin Elmer, Melbourne, Australia) was used to analyze the Hg(II) concentration. Instrument operation parameters are as follows: 1.5 kW (ICP power); alumina injector of 2.0 mm (injector); argon (plasma gas) used with a flow rate of 8.0 L min^−1^; auxiliary gas used with a flow rate of 0.2 L min^−1^; nebulizer gas used with a flow rate of 0.7 L min^−1^; pressure maintained to 3.2 bar; sample uptake rate of 1.5 mL min^−1^; 3 replicates used with an integration time of 10 s and a wavelength of 194.168 nm.

#### 2.1.4. Recommended Procedure for Trace Hg(II) Extraction

A polytetrafluroethylene (PTFE) column (length = 10 cm; diameter = 1 cm) (Merck, Shanghai, China) was used after packing with 50.0 mg of the CNFs@CdS adsorbent. The sample solution (100 mL) of the desired Hg(II) concentration of pH 7.0, maintained using HNO_3_ and NaOH, was percolated through the column bed at a flow rate of 6 mL min^−1^. The flow rate of the sample solution was adjusted with a peristaltic pump (Scenchen, Hebei, China). The adsorbed analyte ions were stripped out using a suitable eluting agent and subsequently analyzed by ICP-OES.

## 3. Results and Discussion

### 3.1. Characterization

**Figure 1:**
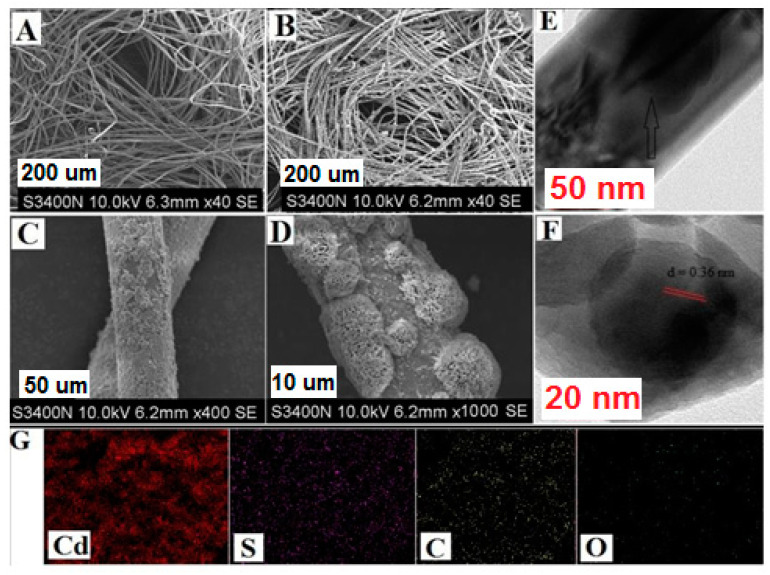
Field emission scanning electron microscope (FESEM) image of: (**A**) nascent cellulose nanofibrils (CNFs); (**B**–**D**) cadmium-sulfide-decorated cellulose nanofibrils (CNFs@CdS) at varying magnifications; (**E**,**F**) high resolution tunneling electron microscopy (HRTEM) images of CNFs@CdS; (**G**) elemental mapping of CNFs@CdS.

**Figure 2:**
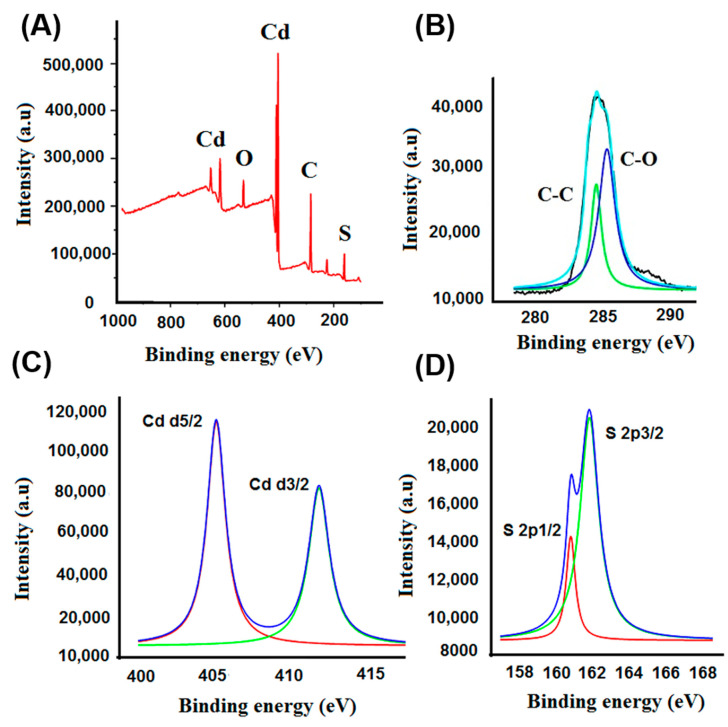
(**A**) XPS survey spectrum of CNFs@CdS; deconvoluted spectra of: (**B**) C 1s; (**C**) Cd 2d; (**D**) S 2p.

The FESEM images in Figure 1A–D show the surface morphology of nascent CNFs and CdS before and after functionalization. The nonfunctionalized CNFs have smooth surfaces (Figure 1A), while the successful decoration of CdS onto CNFs is clearly visible in Figure 1B–D at different magnifications. The flower-shaped nanospheres of CdS were well distributed throughout the fiber’s surface (Figure 1C,D). The CdS microstructure is observed in HRTEM images (Figure 1E,F). From the lattice fringes of CdS (Figure 1F), a 0.36 nm interplanar distance was observed, which corresponded to the (002) plane of CdS, attributed to the hexagonal geometry. The decoration of CdS nanoparticles on the CNFs was well observed by analyzing the elemental composition. The elemental mapping obtained from the FESEM image of CNFs@CdS shows the presence of elemental Cd and S along with C and O atoms on the CNF surface (Figure 1G). XPS data for elemental composition and surface chemical bonding further validate the surface functional groups. The XPS data of the CNFs@CdS adsorbent are shown in Table 1. The XPS survey spectrum of the CNFs@CdS adsorbent, shown in Figure 2A, shows the presence of elemental C and O of the CNFs and Cd and S of the decorated CdS nanoparticles. The deconvoluted peak of C 1s shows the characteristic bonding peaks (C–C and C–O) of cellulose at 284.6 and 285.3 eV, respectively (Figure 2B). The core-level deconvoluted peaks for Cd d5/2 and Cd d3/2 at 405.2 and 412.4 eV (binding energy), respectively, are attributed to the Cd^2+^ ions (Figure 2C). Similarly, Figure 2D shows the deconvoluted peaks of S 2p_1/2_ and S 2p_3/2_ at binding energies of 160.2 and 162.8 eV, respectively, for the S^2−^ ion of the CNFs@CdS adsorbent. The physical characteristic parameters of CNFs@CdS calculated by the BET method were found to be 429.32 m^2^ g^−1^ (surface area), 28.42 nm (pore volume) and 1.69 cm^3^ g^−1^ (pore radius).

### 3.2. Optimization of Solution pH

**Figure 3:**
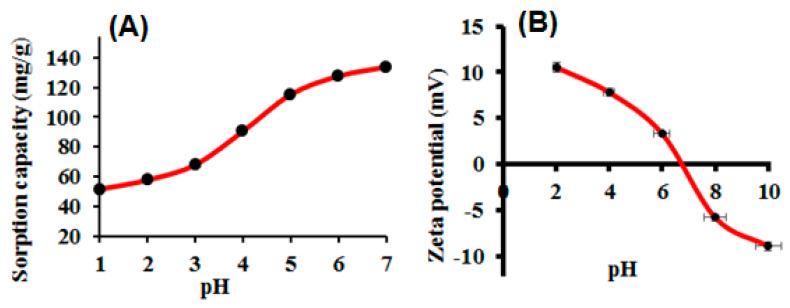
(**A**) Effect of sample pH on the adsorption of Hg(II); (**B**) zeta potential of CNFs@CdS (experimental conditions: flow rate = 6 mL min^−1^; eluent = 5 mL of 0.5 M of HCl; mass of adsorbent = 50.0 mg; Hg^2+^ = 250 mg L^−1^; T = 27 °C).

The solution pH affects the charge distribution generated on the adsorbents due to the protonation and deprotonation of functional groups and metal ion species in aqueous medium. Sample pH plays a crucial role in metal ion adsorption [35]. Herein, we studied the adsorption of Hg(II) in the pH range of 1–7. Basic pH values from 8 to 10 were avoided due to Hg(II) precipitation. To study the effect of pH, a series of model solutions (100 mL) containing 250 mg L^−1^ of Hg(II) was passed through the column using a peristaltic pump. The number of metal ions left in the supernatants was analyzed by ICP-OES to determine the adsorbed amount of Hg(II). The data obtained are presented in Figure 3A. It can be seen that the adsorption of Hg(II) noticeably increases with increasing sample pH in the 1–5 range and remains constant at pH 5–7. At pH 7, the maximum adsorption of Hg(II) was observed and optimized for the remaining experiments. After varying the sample pH values, the surface charge of the CNFs@CdS adsorbent varied drastically. Figure 3B shows the zeta potential of the CNFs@CdS adsorbent. It is observed that, at pH 6.5, the CNFs@CdS surface was negatively charged, which favors the electrostatic interaction with positively charged Hg(II) and enhances the inner-sphere complexation of Hg(II) with CdS at the CNFs@CdS surface, possibly due to the soft–soft acid–base system [36,37]. In conclusion, the complex formation of Hg(II) with sulfur atoms on the CNFs@CdS adsorbent surface is the underlying mechanism of the adsorption phenomenon. The Hg(II) adsorption capacity of CNFs@CdS was found to be 126.0 mg g^−1^ at pH 7.0, which is close to and higher than most of the nanoadsorbents reported for Hg(II) adsorption. Razmi et al. reported the adsorption of Hg(II) (77 mg g^−1^) using eggshell membrane protein doped reduced graphene oxide [38]. Munoz et al. reported the adsorption of Hg(II) by TiO_2_ and titanate nanotubes [39]. Similarly, Basadi et al. reported Hg(II) adsorption onto L-cysteine-functionalized graphene oxide with the maximum adsorption of 98.3 mg g^−1^ [40]. Ozdemir reported Amberlite resin loaded with *Anoxybacillus kestanboliensis* for the adsorption of Co(II) and Hg(II), showing an adsorption capacity of 27.8 mg g^−1^ [41].

### 3.3. Effect of Column Flow Rate

**Figure 4:**
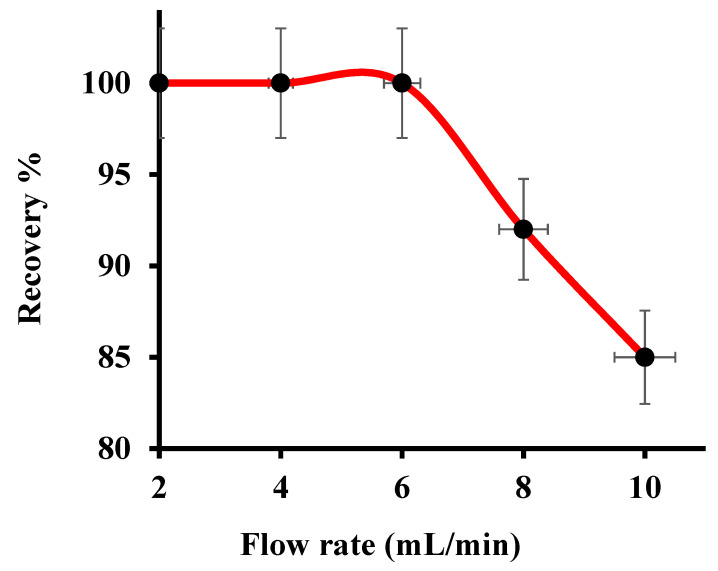
Effect of the column flow rate (experimental conditions: sample pH of 7; eluent =5 mL of 0.5 M of HCl; mass of adsorbent = 50.0 mg; Hg^2+^ = 250 µg mL^−1^; T = 27 °C).

The flow rate of the sample solution affects the retention of metal ions onto the adsorbent bed in the column experiments. To establish an equilibrium between the binding sites and analyte ions, an optimum sample flow is required. To study the effect of sample flow rate on the adsorption of Hg(II), a bench of model solution (volume = 100 mL), containing 250 µg mL^−1^ of Hg(II) and maintained at pH 7.0, was percolated through the CNFs@CdS-packed column at varying flow rates (2 to 10 mL min^−1^). The results are shown in Figure 4. Up to a flow rate of 6 mL min^−1^, the complete recovery of Hg(II) was achieved. This may be attributed to sufficient hydrophilicity of CNFs@CdS caused by the presence of a high number of CNF surface functional groups. At a higher flow rate, the recovery of Hg(II) was gradually decreased to 92–85%. This may be due to the insufficient equilibrium between Hg(II) and the active sites of the CNFs@CdS adsorbent. Hence, an optimum 6 mL min^−1^ volume of the sample flow rate was selected and employed in the remaining experiments.

### 3.4. Eluent Type and Concentration

**Figure 5:**
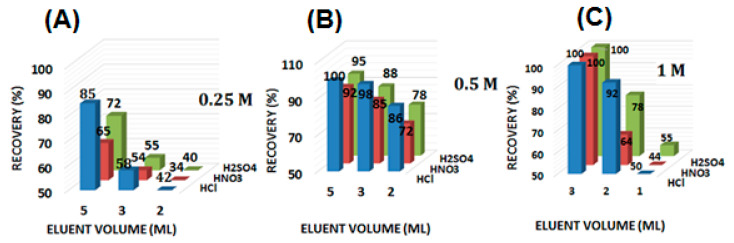
Effect of eluent type, volume and concentration (**A**) 0.25 M; (**B**) 0.5 M; (**C**) 1 M (experimental conditions: sample pH = 6; mass of adsorbent = 50.0 mg; flow rate = 6 mL min^−1^; Hg^2+^ = 250 mg L^−1^; T = 27 °C).

To reuse the SPE column, complete desorption of the sorbed metal ions is necessary. This can be accomplished only by using a suitable stripping agent. Different mineral acids (HCl, HNO_3_ and H_2_SO_4_) with varying volumes (2–5 mL) and concentrations (0.25–1.0 M) were studied (*N* = 3) to optimize the eluent type. The results are shown in Figure 5. High-concentration and high-volume eluents (except 1 M HNO_3_) result in the complete recovery of Hg(II) (recovery >97%). The incomplete elution of adsorbed Hg(II) is observed at lower eluent concentrations (i.e., 0.25 M), with recovery values of less than 90%. Thus, 5 mL of 0.5 M of HCl was optimized as the striping agent to elute the sorbed Hg(II) from the CNFs@CdS-packed column.

### 3.5. Effect of Ionic Strength on Hg(II) Adsorption

Interferences from the alkali and alkaline earth metal, including common heavy metal ions and organic acids, which were inevitably associated with the Hg(II), have been studied. To obtain the results, column studies were performed using a binary mixture solution of metal ion solution and interferents (vol.: 100 mL; Hg(II): 100 µg L^−1^; other metal ions: 10–10^3^-fold). The tolerance limit was set as the ion concentration, which caused a relative error smaller than ±5%. The Hg(II) uptake was then eluted and quantitatively determined by ICP-OES. In conclusion, no noteworthy interferences in the adsorption of Hg(II) were found in the presence of up to a 500-fold concentration of alkali and alkaline earth metal (25-fold for humic and fulvic acids and 100-fold for heavy metal ions), with a Hg(II) recovery of 97–100.4%, indicating the fair selectivity of the CNFs@CdS adsorbent for Hg(II) extraction. The efficient adsorption of Hg(II) on the CNFs@CdS adsorbent could be due to the stable chelation of Hg(II) with the adsorbent. It is observed that following the optimum experimental conditions, the quantitative recovery of Hg(II) from coexisting ions is feasible.

### 3.6. Preconcentration Limit and Breakthrough Studies

**Figure 6:**
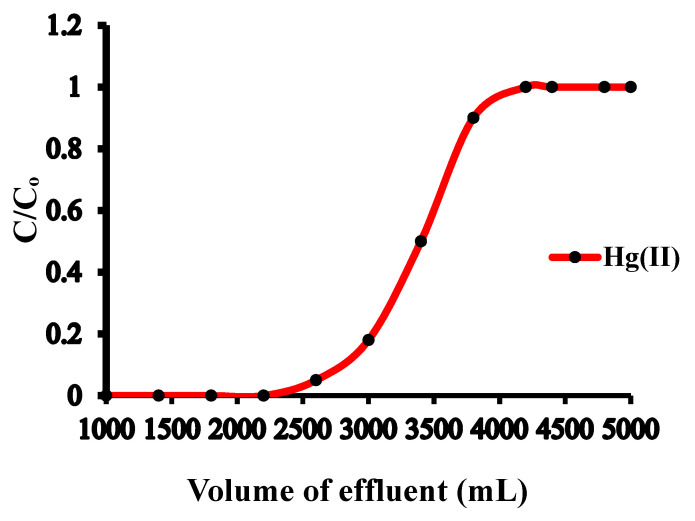
Breakthrough curve for the Hg(II) uptake.

Direct analysis of Hg(II) level in environmental samples is challenging due to the trace concentrations of Hg(II) by spectral interferences caused by matrix interference. A sample preparation technique is necessitated to enhance the analyte concentration and eliminate the interferent. To study the lower limit of the analyte that can be quantitatively determined, a series of model solutions with varying sample sizes containing 1.0 µg of Hg(II) and maintained at pH 6.0 were percolated through the CNFs@CdS-packed column at a flow rate of 6 mL min^−1^. The sorbed Hg(II) was then eluted using 5 mL of 0.5 M of HCl (stripping agent), and the amount of Hg(II) in the eluent was quantitatively determined by ICP-OES. Table 2 presents the obtained data. It was observed that the quantitative recovery of Hg(II) was achieved within a sample volume of 2800 mL, while upon increasing the sample volume to 2900–3000 mL, the percent recovery of Hg(II) noticeably decreased to 92%. A high preconcentration limit of 0.36 µg L^−1^ was obtained with a preconcentration factor of 580. Such a high preconcentration factor was necessitated for the column preconcentration of trace metal ions and was found comparable and better than other reported nanoadsorbents [38,40,41]. To determine the breakthrough volume, 5 L of 2.0 mg L^−1^ of Hg(II) solution, maintained at pH 6.0, was continuously percolated through the column at a flow rate of 6.0 mL min^−1^. At varying time intervals, the fractions of effluent were gathered, and the concentration of Hg(II) in the fractions was determined. The effluent volume at which the concentration of Hg(II) was 3–5% of the initially loaded metal concentration corresponded to the breakthrough volume and was found to be 2900 mL. Figure 6 shows the breakthrough curves for Hg(II), and the corresponding breakthrough capacity obtained for the CNFs@CdS adsorbent was 126.0 mg g^−1^.

### 3.7. Analytical Methods’ Validation

Analytical method validation is a subject of consideration irrespective of the suitability of the developed procedure for providing useful data. Following the optimum experimental conditions (sample pH = 7; flow rate = 6 mL min^−1^; eluent volume = 5 mL; adsorbent amount = 50 mg), the calibration plot for Hg(II) analysis was obtained in the range of 0.2–100 µg L^−1^ of Hg(II), with a good correlation coefficient (R^2^ = 0.9998). The limit of detection (LOD) and the limit of quantification (LOQ), obtained as the concentration of analyte corresponding to three and ten times the standard deviation of 11 runs of the mean blank signal, were found to be 0.06 and 0.2 µg L^−1^, respectively [38,42]. The detection limit method is suited for trace metal concentration and comparable with previously reported analytical methods using nanoadsorbents [40,41]. The relative standard deviation (RSD), characterizing the precision of the method, evaluated for ten replicate samples containing 5 µg L^−1^ of Hg(II), was found to be 2.4%. The proposed method was validated by analyzing standard reference materials (SRM 1641d), and the results are shown in Table 3. The closeness of the measured value is in good agreement with the certified values, indicating the accuracy of the developed method. In addition, the spiking analysis with two levels of Hg(II) concentration was carried out using different environmental water samples such as tap water, industrial wastewater and river water samples (Table 4). With a 95% confidence limit, the spiked amount of Hg(II) was recovered satisfactorily and the mean percentage recoveries ranged from 99.3% to 101%, with a relative standard deviation (RSD) in the range of 1.2–3.8%.

## 4. Conclusions

The successful in situ growth of CdS nanoparticles onto the CNFs surface has been reported. The prepared material shows an excellent affinity for Hg(II) extraction compared to existing nanoadsorbents. The proposed method shows a high preconcentration limit of 0.36 µg L^−1^ with a preconcentration factor of 580. The proposed methodology was validated by determining the Hg(II) concentration in the standard reference material and analyzing the spiked amount of Hg(II) in real samples (recovery > 97%; RSD < 5%). The proposed method eliminated the matrix interferences and was successfully employed to monitor the Hg(II) contamination level in real water samples. Our findings may open new avenues to other 2D metal chalcogenides/nanomaterials as potential nanoadsorbents to be used in sample preparation techniques to monitor the trace-level concentrations of environmental water pollutants.

## Figures and Tables

**Table 1:** Elemental data obtained from the XPS survey of the cadmium-sulfide-decorated cellulose nanofibrils (CNFs@CdS) adsorbent.

Element	Peak Position (eV)	Height cps	Atomic %	Area(P) cps	FWHM
C 1s	284.82	30,985.25	61.84	84,758.97	2.31
O 1s	531.81	10,067.49	11.89	33,367.52	3.00
S 2p	161.71	12,465.10	11.5	29,568.62	2.14
Cd 3d5	404.75	96,527.63	10.78	208,022.13	2.10

X-ray photoelectron spectroscopy (XPS); cadmium-sulfide-decorated cellulose nanofibrils (CNFs@CdS); full width at half maximum (FWHM).

**Table 2:** Preconcentration and breakthrough studies using CNFs@CdS adsorbent (column parameters: sample = pH 7; eluent volume = 5 mL; flow rate = 6 mL min^−1^; adsorbent amount = 25 mg).

Preconcentration Studies				Breakthrough Studies	
**Sample Volume (mL)**	Hg(II) (μg L^−1^)	Preconcentration Limit (μg L^−1^)	Preconcentration Factor	Breakthrough Volume (mL)	Breakthrough Capacity (mg g^−1^)
1000	1.00	1.00	200	2900	126.0
1500	0.67	0.67	300		
2000	0.50	0.50	400		
2500	0.40	0.40	500		
2800	0.36	0.36	560		
3000	0.33	-	-		

**Table 3:** Analytical method validation by analyzing the standard reference material (SRM).

Samples	Certified Values (μg g^−1^)	Values Found by the Proposed Method (μg g^−1^) ± Standard Deviation ^a^	*t*-Test ^b^
SRM 1641d	1.56 ± 0.02	1.53 ± 0.52	2.37

^a^ Mean value for *N* = 3; ^b^ at the 95% confidence level.

**Table 4:** ICP-OES determination of Hg(II) concentration in real samples after preconcentration and solid-phase extraction.

Samples	Spiked Amount (μg)	Amount Found (μg L^−1^) ± Standard Deviation ^a^	Recovery Percent (RSD) ^c^	Value of *t*-Test; at 95% Confidence Level ^d^
Tap water	0	ND ^b^	-	-
	3	3.01 ± 0.53	100.3 (2.46)	0.952
	5	4.98 ± 0.71	99.6 (2.24)	1.273
	0	4.8	-	-
Industrial wastewater	3	7.78 ± 0.87	99.3 (3.80)	1.125
River water	5	9.82 ± 0.76	100.4 (2.39)	2.041
	0	1.13 ± 0.64	-	1.194
	3	4.12 ± 0.25	99.7 (1.29)	1.918
	5	6.18 ± 0.53	101 (2.17)	2.231

^a^ Mean value for *N* = 3; ^b^ not detected; ^c^ relative standard deviation; ^d^ *t*_critical_ = 4.303.
